# Happy Childhood

**DOI:** 10.14797/mdcvj.1216

**Published:** 2023-03-07

**Authors:** Philip Alexander

**Affiliations:** 1 College Station, Texas, US

## Abstract

Philip Alexander, MD, is a native Texan, retired physician, and accomplished musician and artist. After 41 years as an internal medicine physician, Dr. Phil retired from his practice in College Station in 2016. A lifelong musician and former music professor, he often performs as an oboe soloist for the Brazos Valley Symphony Orchestra. He began exploring visual art in 1980, evolving from pencil sketches—including an official White House portrait of President Ronald Reagan—to the computer-generated drawings featured in this journal. His images, which first appeared in this journal in the spring of 2012, are his own original creations.

If you would like to see your art published in the *Methodist DeBakey Cardiovascular Journal*, submit your creation online at journal.houstonmethodist.org as a “Humanities” entry.

**Image 1:**
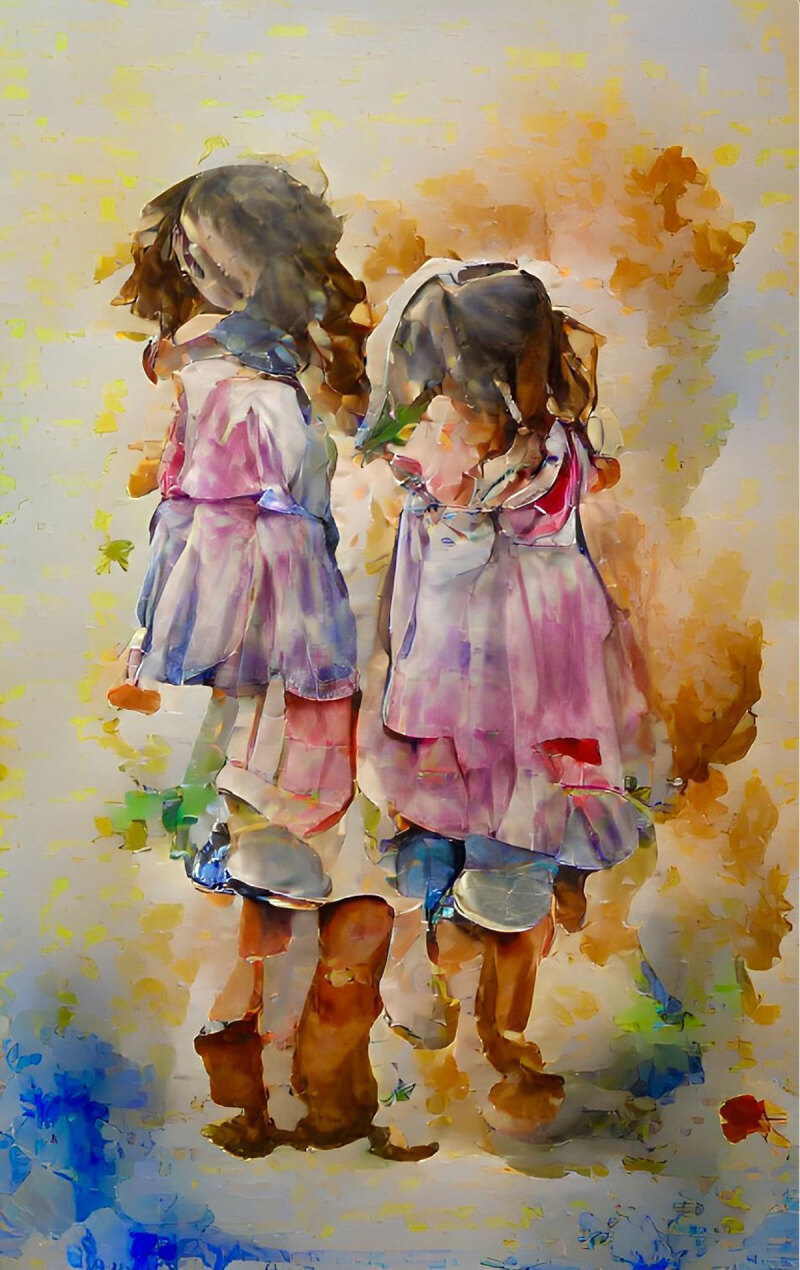
Sisters.

**Image 2:**
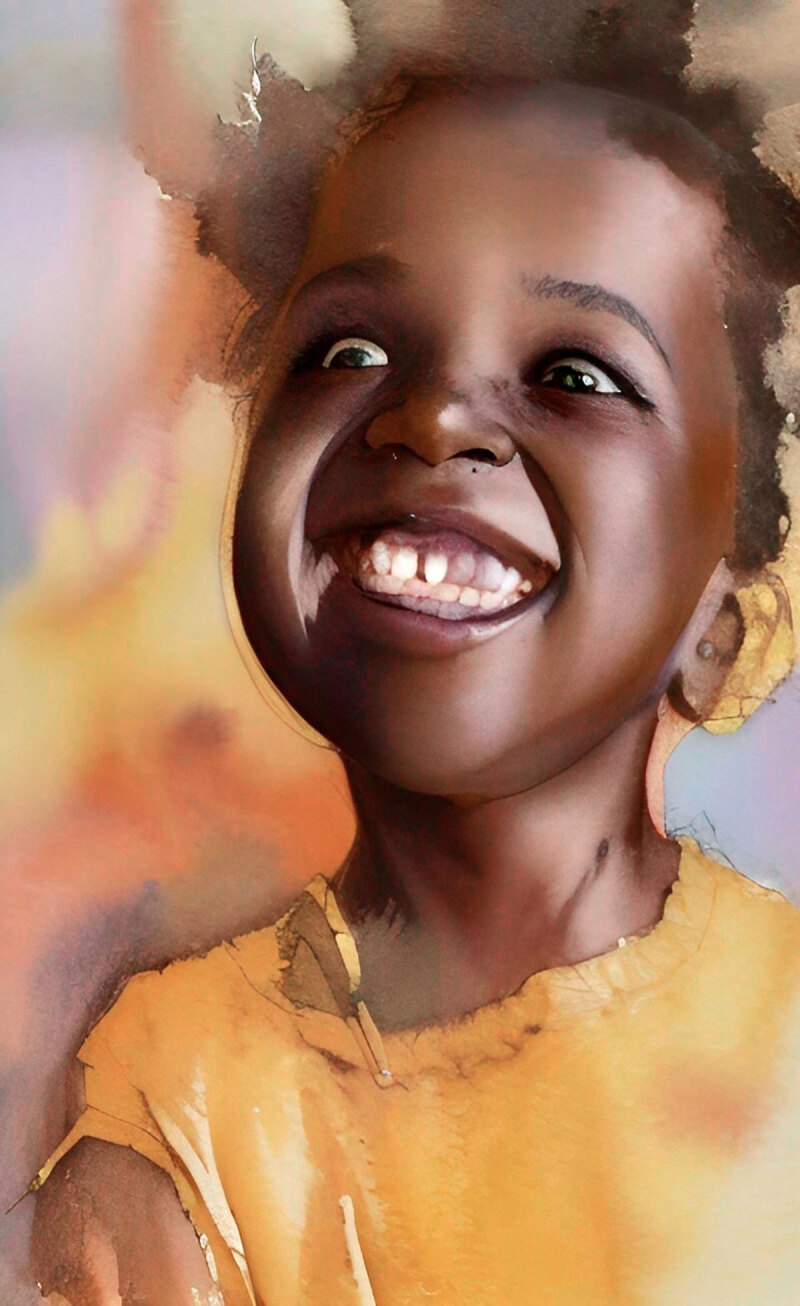
What Happy Looks Like!

